# New Work for the State Board of Health

**Published:** 1881-07

**Authors:** 


### Editorial.

### NEW WORK FOR THE STATE BOARD OF HEALTH.

IF any intelligent physician ever entertained a doubt as to the necessity of a State Board of Health, such skepticism must have vanished in the light of facts constantly brought to his attention. A spirit of criticism—more potent than the will of Jupiter—imposed such Herculean labors upon this Board, as would make it well deserving of immortal memory by the completion of even a twelfth part of the task. The problems which thus confront it, vary in character from the repression of quacking in its hydra headed forms, to the cleansing of the Augean stables of our public streets. We would not, therefore, attempt any multiplication of these labors by idle suggestions, but simply call attention to an evil which might be readily remedied. It is well known that this State supports two institutions for the blind, where the condition of that unfortunate class is materially improved by the excellent care of trained teachers. But it is *not* generally known that among the pupils thus received as incurably blind, there are not a few who might be made to see much better, if not be entirely cured. Frequently they come from remote districts, without ever having consulted any one, except a local doctor, who glanced at their eyes and jumping at the conclusion that nothing could be done, pronounced the sentence of "incurable." It never occurred to him, perhaps, that an artificial pupil could be made, or a cataract extracted, and as a consequence these children spend one or more years of their lives in the pursuit of knowledge, under difficulties well nigh insurmountable. Meanwhile, the patience of the good teachers is severely taxed, the cost of the institution increased and the individual deprived of the pleasures and comforts which come with the sense of vision. This could easily be obviated, if the State Board of Health possessed the power of appointing some competent person—or, perhaps, it would as a body undertake—to examine the applicants at these institutions and determine exactly what ones were to be admitted and what ones were yet curable. In this way could a considerable yearly saving be made for the State, and in this way, too, would many a one be led to seek that aid which might transform a semi-mendicant into a self-reliant, self-supporting person.

